# PTMsnp: A Web Server for the Identification of Driver Mutations That Affect Protein Post-translational Modification

**DOI:** 10.3389/fcell.2020.593661

**Published:** 2020-11-10

**Authors:** Di Peng, Huiqin Li, Bosu Hu, Hongwan Zhang, Li Chen, Shaofeng Lin, Zhixiang Zuo, Yu Xue, Jian Ren, Yubin Xie

**Affiliations:** ^1^Precision Medicine Institute, The First Affiliated Hospital, School of Life Sciences, Sun Yat-sen University, Guangzhou, China; ^2^State Key Laboratory of Oncology in South China, Cancer Center, Collaborative Innovation Center for Cancer Medicine, Sun Yat-sen University, Guangzhou, China; ^3^Key Laboratory of Molecular Biophysics of Ministry of Education, Hubei Bioinformatics and Molecular Imaging Key Laboratory, Center for Artificial Intelligence Biology, College of Life Science and Technology, Huazhong University of Science and Technology, Wuhan, China

**Keywords:** protein post-translational modification, genetic mutations, Bayesian hierarchical model, driver genes, disease

## Abstract

High-throughput sequencing technologies have identified millions of genetic mutations in multiple human diseases. However, the interpretation of the pathogenesis of these mutations and the discovery of driver genes that dominate disease progression is still a major challenge. Combining functional features such as protein post-translational modification (PTM) with genetic mutations is an effective way to predict such alterations. Here, we present PTMsnp, a web server that implements a Bayesian hierarchical model to identify driver genetic mutations targeting PTM sites. PTMsnp accepts genetic mutations in a standard variant call format or tabular format as input and outputs several interactive charts of PTM-related mutations that potentially affect PTMs. Additional functional annotations are performed to evaluate the impact of PTM-related mutations on protein structure and function, as well as to classify variants relevant to Mendelian disease. A total of 4,11,574 modification sites from 33 different types of PTMs and 1,776,848 somatic mutations from TCGA across 33 different cancer types are integrated into the web server, enabling identification of candidate cancer driver genes based on PTM. Applications of PTMsnp to the cancer cohorts and a GWAS dataset of type 2 diabetes identified a set of potential drivers together with several known disease-related genes, indicating its reliability in distinguishing disease-related mutations and providing potential molecular targets for new therapeutic strategies. PTMsnp is freely available at: http://ptmsnp.renlab.org.

## Introduction

Large-scale genome sequencing has uncovered a complex landscape of genetic mutations in multiple patient populations. A major goal of these sequencing projects is to characterize a few disease-related mutations from the majority of neutral passenger mutations. Currently, the most commonly used strategy to prioritize mutations is the frequency-based approach, such as MutSigCV (Lawrence et al., 2013), MuSiC (Dees et al., 2012), and other methods (Youn and Simon, 2011). These tools can reveal a number of potential driver genes that carry recurrent mutations in a given disease cohort. However, the known driver genes identified from those frequency-based strategies are not sufficient to explain the diverse mechanisms of disease progression. Therefore, several approaches that not only consider recurrent mutations but also combine other functional features, such as evolutionary conservation (Reva et al., 2011), known pathway annotation (Wendl et al., 2011) and protein-protein interaction networks (Vandin et al., 2011; Ciriello et al., 2012), have been proposed.

Among those functional features, one of the most critical factors that can be used in driver gene identification is protein post-translational modifications (PTMs). As key mechanisms to increase proteomic diversity, PTMs can regulate almost all physiological and biochemical processes in mammalian cells. Thus, genetic mutations that occur specifically around the PTM sites (also known as PTM-related mutations) may potentially alter protein functions and disturb regulatory pathways *in vivo*, leading to the development of certain serious diseases, such as cancers. A previous study has reported that mutation of SUMO-conjugated sites in androgen receptor (AR) may result in an increase of AR transcriptional activity, and hence promoting cell proliferation and hypoxia-induced angiogenesis in Prostate cancer (Lin et al., 2004). Meanwhile, experiments have also shown that oncogenic variants altering S768 phosphorylation of EGFR increase its catalytic activity, and S768I mutation can drive tumorigenesis by disrupting EGFR autophosphorylation and rewiring downstream signaling pathways (Huang L. C. et al., 2018). In addition to cancer, Martin et al. have reported that the G553E mutation on huntingtin (HTT) protein can abrogate its post-translational myristoylation and induce cellular toxicity of the protein in cellulo, consequently causing Huntington disease (Martin et al., 2018).

In light of the significant impact of PTM-related mutations on human diseases, several databases have been developed to curate mutations that may potentially affect PTMs. For example, dbPTM collected a subset of PTM-disease associations based on disease-associated non-synonymous SNPs from dbSNP in its 2019 updated version (Huang et al., 2019). Similarly, PhosphpSitePlus provided PTMVar dataset to characterize PTMs that overlap with disease-associated genetic variants and polymorphisms (Hornbeck et al., 2015). Using a similar strategy, other databases such as iPTMnet (Huang H. et al., 2018), PRISMOID (Li et al., 2020), and PTM-SNPs (Kim et al., 2015) were also reported in recent publications. In considering the false positive errors that introduced by the direct mapping of disease-related mutations to PTM sites when deriving disease-related PTM mutations, several studies using predictive tools to extract PTM-related mutations were proposed. For instance, ActiveDriver revealed a set of candidate cancer driver genes harboring mutation hotspots proximal to known phosphorylation, acetylation and ubiquitination sites that may cause the dysfunction of PTM-related mechanisms (Reimand and Bader, 2013; Reimand et al., 2013; Narayan et al., 2016). Besides, MIMP is a machine learning method to predict whether single-nucleotide variants (SNVs) can disrupt existing phosphorylation sites or create new sites (Wagih et al., 2015). Using the MIMP method, ActiveDriverDB is established for collecting human disease mutations and genetic variants that may potentially alter four types of PTMs (Krassowski et al., 2018). In addition, AWESOME utilized 20 PTM prediction tools to predict whether a SNP could change the PTMs level of six common PTM types in a specific protein (Yang et al., 2019). Besides, Simpson et al. developed DeltaScansite to assess the impact of mutations in the flanking regions of phosphosites (Simpson et al., 2019).

Although these reported methods have provided abundant resources of PTM-related mutations, limitations are still existing. First of all, the current methods carried out mutation analysis for one or a few common PTM types, and most other PTM types cannot be covered, thus losing a large amount of PTM-related mutation information. Secondly, most of methods (except ActiveDriver) only consider the impact of mutations on PTM sites alone, and are not associated with specific disease phenotypes, which may preserve a lot of passenger mutations that play a neutral role in disease development. Meanwhile, ActiveDriver only focused on cancer somatic mutations affecting PTMs, but did not extend to other serious diseases. Finally, previous studies mainly developed a database to curate PTM-related mutations obtained by their computational methods for user search, there is still no web-based tool available to annotate rare mutations in new disease research by PTM function. Therefore, existing computational tools are insufficient to assist PTM-mediated disease driver identification, an efficient and easy-to-use mutation analysis tool to discover disease driver mutations that affect a variety of PTM types are in great need to investigate the pathogenesis and development of multiple serious diseases.

In this paper, we introduce PTMsnp, a web server that implements a Bayesian hierarchical model to detect driver proteins with significant PTM-related mutations. PTMsnp has integrated 4,11,574 modification sites from 33 different types of PTMs and 1,776,848 somatic mutations of 33 cancer types. From PTMsnp, one can easily identify significantly PTM-mutated proteins (also known as driver genes) across different cohorts from TCGA. In addition, users can upload their own mutation resources, e.g., cohorts from genome-wide association studies (GWASs), to obtain significantly PTM-mutated proteins as well as potential disease-related mutations that significantly affect PTM status. In order to further evaluate the functional importance of PTM-related mutations, we also integrated multiple computational predictive programs for variant interpretation and clinical classification. To illustrate the functionality of PTMsnp, we applied it to TCGA cancer cohorts and a GWAS dataset of type 2 diabetes cohorts. Several known disease-related genes were successfully identified by PTMsnp, demonstrating that it is practicable to discover putative disease-related genes and hypothesize how they biochemically function in disease development.

## Materials and Methods

### PTMsnp Algorithm

To identify proteins with a significantly high number of PTM-related mutations, we first converted the coordinates of genetic mutations from the genomic level to the protein level using ANNOVAR (Wang et al., 2010). For analysis, only non-synonymous SNVs that did not create a premature stop codon or remove the existing stop codon were retained. According to previously published literatures (Reimand and Bader, 2013; Reimand et al., 2013; Narayan et al., 2016; Chen et al., 2018), the protein sequence flanking the central PTM site within seven residues was taken as the PTM motif region. The same type of PTM motif regions in the same protein were then merged to create a modification region. Correspondingly, the remaining sequences were merged separately and denoted as background regions. The frequency of each non-synonymous SNV located in the modification region and the background region were separately calculated.

We assumed that, in the patient group, mutations located in the PTM motif regions would probably damage the modification process, thereby influencing protein functions via PTM-related pathways. If such mutations are highly correlated with a given disease lesion, they will probably undergo strong positive selection; therefore, unexpectedly high mutation rates will be observed in these regions. According to this assumption, we developed the following Bayesian hierarchical model to compare the mutation rate between modification regions and background regions.

First, for a given protein, let *Y_1_, Y_2_, …, Y_*k*_* represent the count of mutations at each position in the modification region, and let *Y_*k*__+__1_, Y_*k*__+__2_, …, Y_*n*_* be the same count in the background region. We then modeled the observed counts Y by a Poisson distribution as shown in Equations 1 and 2, where *λ_1_* and *λ_2_* are the mutation rates of the modification region and the background region, respectively.

(1) $$Y_{\text{i}}∼P\,o\,s\,s\,i\,o\,n\,\left(\lambda_{1}\right)\;i=1,2\,\ldots ,k$$

(2) $$Y_{\text{i}}∼P\,o\,s\,s\,i\,o\,n\,\left(\lambda_{2}\right)\;i=k+1,k+2,\ldots ,n$$

Since the mutation rate may vary markedly in different positions, a prior distribution was applied to *λ_1_* and *λ_2_* to capture such fluctuation. As stated in the theory of probability, a gamma distribution is the conjugate prior to the Poisson distribution. Therefore, two gamma distributions with different shape parameters α and scale parameters β were used to describe the distribution of *λ_1_* and *λ_2_* in Eqs 3 and 4.

(3) $$\lambda_{1}∼G\,a\,m\,m\,a\,\left(\alpha_{1},\beta_{1}\right)$$

(4) $$\lambda_{2}∼G\,a\,m\,m\,a\,\left(\alpha_{2},\beta_{2}\right)$$

To test the difference between the mutation rates of the background and those of the modification regions, a variable of interest might be the relative mutation rate, which is defined as *R = λ_1_/λ_2_*. Given that, a statistical hypothesis was raised as shown below.

(5) $$H_{0}:R\leq 1$$

(6) $$H_{1}:R>1$$

The *p*-value under the null hypothesis can therefore be calculated from the marginal distribution of *R* given the observed data *Y*. A Markov chain Monte Carlo (MCMC) method was applied to infer such distribution. To control false positives, the Benjamini-Hochberg procedure is applied to each *p*-value. If the corrected *p*-value for a given protein is lower than the significance level, i.e., 0.05, we identify it as a potential disease driver (Supplementary Methods).

### Database for PTM Sites and Mutations

PTM sites of human proteins were retrieved from the dbPTM (2019 update), iPTMnet (November 2019) database and manually collected from published literatures in PubMed. To unify the heterogeneity of data collected from different sources and ensure site accuracy, we mapped the reported modification sites to UniProtKB protein entries and used sequence comparison to correct the original data information and retain protein isoforms. Each mapped PTM site is attributed with a corresponding literature (PubMed ID) and source.

Somatic mutations were downloaded from the data portals of TCGA (18 July 2019)^1^. To construct an intact set of somatic mutations, mutations generated by four different variant calling workflows were merged and duplicated sites were removed. The ANNOVAR program was applied to annotate the functional consequence of all mutation sites. Only non-synonymous SNVs that did not create a premature stop codon or remove the existing stop codon were retained in our database.

### The Processing of WTCCC T2D Dataset

The Wellcome Trust Case Control Consortium (WTCCC) Type 2 Diabetes (T2D) datasets consisted of individual–level genotypes called by BRLMM and Chiamo (The Wellcome Trust Case Control Consortium, 2007) were collected in this study. All SNPs were mapped to GRCh38 (hg38) genomic coordinates according to their RSIDs to facilitate the annotation of SNPs and proteins. Unmapped RSIDs was discarded. For genotypes called by BRLMM, calls with score < 0.5 were retained. For the Chiamo data, the recommended probability threshold for inclusion is > 0.9. After excluding low-quality samples or calls, the valid calls derived from two calling methods are intersected to obtain the reliable genotypes of all samples in T2D. Finally, all genotypes are processed into VCF files and used as input for PTMsnp.

## Results

### Data Statistics of PTM Sites and Mutations

To assist the functional studies of cancer mutations, PTMsnp provides a database of known PTM sites and somatic mutations. PTM sites of human proteins are mainly derived from dbPTM (2019 update), a database that manually curated PTM peptides from the published literatures and integrated experimentally verified PTM sites from 30 available PTM-related resources such as PhosphoSitePlus (Hornbeck et al., 2015), dbPAF (Ullah et al., 2016), UniProtKB (Boutet et al., 2007), PLMD (Xu et al., 2017), and Phospho. ELM (Dinkel et al., 2011) etc. We also collected additional PTM modification sites in iPTMnet, as well as manually curated from published literatures in PubMed. After strict data correction and filtering, a total of 4,11,574 PTM sites, covering Phosphorylation, Ubiquitination, Acetylation, Methylation, Sumoylation, Malonylation, O(N/C/S)-linked Glycosylation, S-nitrosylation, Glutathionylation, Succinylation, Nitration, Palmitoylation, Myristoylation, Hydroxylation, Crotonylation, Sulfation, Farnesylation, Geranylgeranylation, Gamma-carboxyglutamic acid, Pyrrolidone carboxylic acid, Citrullination, Glutarylation, Amidation, Carbamidation, Oxidation, GPI-anchor, Lipoylation, Neddylation, Carboxylation, and Pyruvate, were curated in our web server. On the other hand, somatic mutations downloaded from the data portals of TCGA were processed to retain non-synonymous SNVs, and finally, 1,776,848 non-synonymous SNVs across 33 cancer types (UCEC, SKCM, COAD, LUAD, STAD, LUSC, BLCA, BRCA, HNSC, GBM, CESC, OV, READ, LIHC, LGG, ESCA, PAAD, PRAD, KIRC, SARC, KIRP, ACC, LAML, UCS, THCA, DLBC, CHOL, THYM, MESO, TGCT, KICH, PCPG, and UVM) were collected in PTMsnp (Supplementary Table S1).

### Web Server Description

**FIGURE 1:**
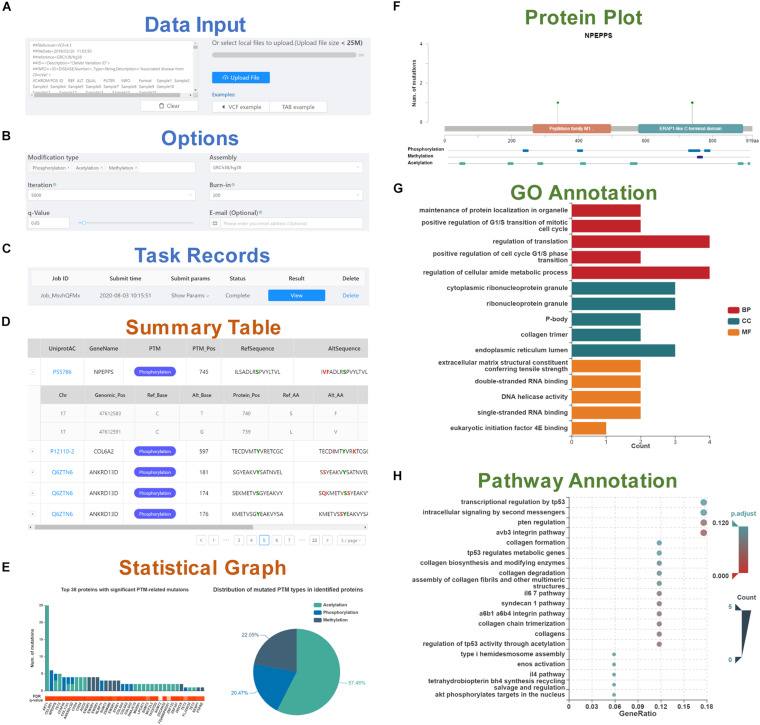
A schematic workflow of the PTMsnp web server. **(A)** Data input section. **(B)** Six options set for the PTMsnp program. **(C)** Task records to monitor the running task and view the results. The result page consists of five parts, including **(D)** A summary table of significantly PTM-mutated proteins. **(E)** The statistical graphs of significant PTM-related mutations and mutated PTM types in identified proteins. **(F)** The mutation sites on the protein sequence and its known functional domains. **(G)** GO annotation of identified proteins. **(H)** KEGG pathway enrichment of identified proteins.

To start PTMsnp, genetic mutations in standard VCF or TAB format need to be inputted in the text area or uploaded via the file selection box (Figure 1A). An intact set of somatic mutations from the cancer cohort of TCGA is integrated into the database, and users can also select a cancer type of interest to start analysis. Before calculation, several options, including PTM type, genome assembly version, iteration and burn-in times for the MCMC process, and *q*-value threshold should be set for the PTMsnp program (Figure 1B). Besides, users can enter email address to receive email notifications after the calculation is completed. After the submission of an analysis task, a new record will be added to the task monitoring bar at the bottom of the submit page (Figure 1C). When a task status is displayed as “complete,” the user can click the “view” button to open the result page.

The result page consists of five interactive tables and graphs. The significantly PTM-mutated proteins that may drive the progression of diseases are outputted as a summary table (Figure 1D), supporting interactive operations such as filtering and sorting by cancer type, UniProt accession number, protein name and modification type. Each protein is directly linked to the UniProt database according to its accession number for details. The PTM-related mutations located in these proteins can be expanded or collapsed by click each protein record. Original information of PTM-related mutations such as base changes and genotypes are retained, as well as allele frequency obtained from ExAC database. Meanwhile, we scored the pathogenic level of each PTM-related mutation from 0 to 7 by counting the deleterious results of seven functional predictors [SIFT (Kumar et al., 2009), LRT (Chun and Fay, 2009), MutationTaster (Schwarz et al., 2010), MutationAssessor (Reva et al., 2011), FATHMM (Shihab et al., 2013), MetaSVM, and MetaLR (Dong et al., 2015)] curated in the dbNSFP database (Liu et al., 2016). Besides, InterVar (Li and Wang, 2017), and Clinvar (Landrum et al., 2018) are also integrated for clinical interpretation of PTM-related mutations by the ACMG/AMP 2015 guideline (Richards et al., 2015) and known disease association, respectively. For visualization, the distribution of significant PTM-related mutations and mutated PTM types in identified proteins are plotted in a bar graph and a pie chart (Figure 1E). In addition, for each identified protein, the mutation sites and known PTM sites together with their functional domains are presented in a schematic biological sequence diagram, where users can freely add or remove PTM tracks (Figure 1F). Moreover, to gain further insights into the protein function, we performed Gene Ontology (GO) and pathway enrichment analysis using the clusterProfiler package in R (Yu et al., 2012). The analysis results were illustrated in bar graphs (Figure 1G) and bubble plots (Figure 1H). All visualization diagrams are available in publication quality for download.

### PTMsnp Identifies Known Cancer Genes With Significantly PTM-Related Mutations

**FIGURE 2:**
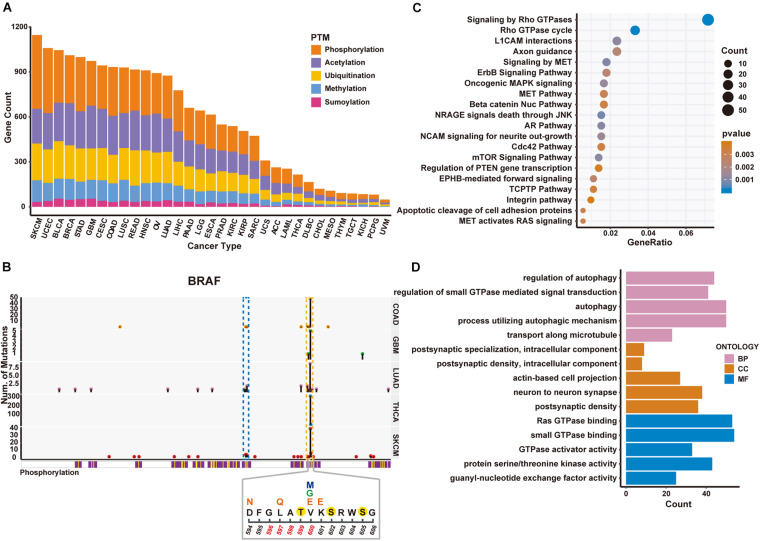
Significantly mutated proteins identified in TCGA cancer cohorts regarding 5 PTM types. **(A)** Number of significantly PTM-mutated genes across five PTM types identified in different cancers. **(B)** Schematic diagram of mutations and protein phosphorylation modification regions within BRAF gene in five cancer types. Upper panel shows the number of mutated samples per position. The blue and yellow dashed boxes represent the P-loop and activation loop on the BRAF protein, respectively. The lower panel shows the mutation and phosphorylation within 594–606 region of the BRAF protein in SKCM. Positions 596–600 are the activation segment. Above the position coordinates is the amino acid sequence. The phosphorylated amino acids are marked with a yellow solid circle. Altered amino acid after mutation is above the original sequence. V600 has three different mutation forms, marked with different colors. **(C)** The enriched pathways of PTM-mutated proteins in SKCM. **(D)** The enriched GO terms obtained from the identified PTM-mutated proteins in SKCM.

**FIGURE 3:**
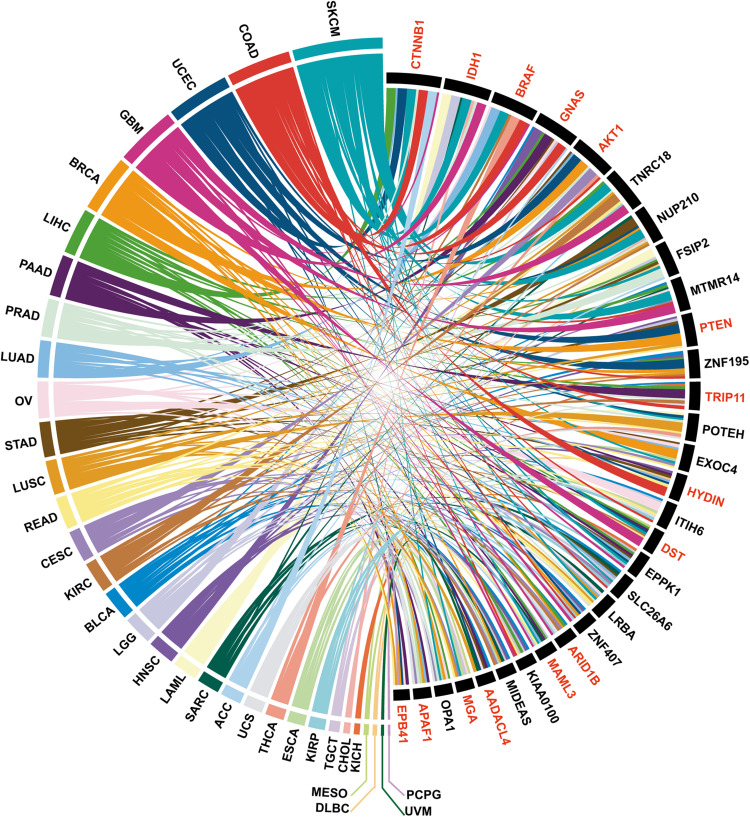
The top 30 PTM-mutated genes identified in more than 7 cancer types, among which the known cancer genes are indicated in red.

To demonstrate how PTMsnp can be used for cancer driver genes detection, we first applied PTMsnp to analyze the somatic mutations from TCGA cohorts across 33 different cancer types. We selected five PTM types, including phosphorylation, acetylation, ubiquitination, methylation and sumoylation, with the largest number of modification sites to analyze the significant PTM-related mutations in cancer patients. PTMsnp identified 9,359 genes with significantly unexpected numbers of PTM-related mutations (*P* = 0.01, Figure 2A and Supplementary Tables S2, S3). Known cancer genes collected from the Cancer Gene Census (CGC) (Sondka et al., 2018), Network of Cancer Genes (NCG 6.0) (Repana et al., 2019), ONGene (Liu et al., 2017) as well as TSGene 2.0 (Zhao et al., 2016) database are significantly enriched (*n* = 2,064, *P* = 1.455 × 10-8, Fisher’s exact test, Supplementary Table S4) in our result. Approximately, one–fourth of the identified genes (*n* = 2,256) contained significant PTM-related mutations in multiple cancer types. Of which, 660 genes were well-known cancer genes, such as CTNNB1, IDH1 (Figure 3). These results showed that the significantly PTM-mutated genes identified by PTMsnp may have a broad and important functional impact in the cancer driving mechanism.

Moreover, we found that PTMsnp identified the largest number of significantly PTM-mutated genes in Skin Cutaneous Melanoma (SKCM, Figure 2A). The BRAF gene ranked first by the number of PTM-related mutations in SKCM and harbored multiple significant PTM mutations in several cancer types (Figure 3). BRAF, also known as serine/threonine-protein kinase B-Raf, can phosphorylate MAP2K1 and thereby activates the MAP kinase signal transduction pathway in living cells. Mutations that activate BRAF functions are present in over 60% of all melanomas (Davies et al., 2002). Studies have shown that BRAF mutations are clustered within the P-loop and activation segment of the kinase domain (Pratilas et al., 2012; Figure 2B). These mutations destabilize the interaction between P-loop and the activation segment, which normally locks the kinase in its inactive state until the activation loop is phosphorylated. Consistently, our method has identified a hotspot mutation at V600 of BRAF can significantly altered the modification level of three phosphorylation sites, namely Thr599, Ser602, and Ser605. One of these phosphorylation sites, Thr599, is located in the activation loop and believed to be functional in regulating the activation of BRAF (Lavoie and Therrien, 2015; Kiel et al., 2016). Three other mutations, including D594N, L597Q, and K601E, are also observed to potentially affect the phosphorylation at Thr599 (Figure 2B). Existing studies have confirmed that these mutations activate the MAPK pathway in melanoma and are associated with sensitivity to MEK inhibitor drug therapy (Dahlman et al., 2012; Wu et al., 2017). In view of these evidences, we hypothesized that the proto-oncogene BRAF is activated by mutations promoting the phosphorylation of its activation loop, implying the feasibility of applying PTMsnp to analyze cancer mutations from the perspective of affecting PTM modification.

Furthermore, we performed pathway analysis on the identified driver genes using MSigDB C2 Canonical pathways (Liberzon et al., 2015) to explore the biological system driven by PTM-related mutations in SKCM (Figure 2C). The top 20 enriched pathways were known to regulate cell proliferation, migration, differentiation, apoptosis, and cell motility, therefore highlighted altered PTM level may be an important hallmark of cancers (Hanahan and Weinberg, 2011). Similar results were also observed in GO enrichment analysis (Figure 2D). These driver genes are enriched in cellular processes such as autophagy whose dysregulation has been linked to many human pathophysiologies including cancer (Chen and Klionsky, 2011; Jiang and Mizushima, 2014). All the above results demonstrated the functional importance of PTM functions in cancer development. Taken together, we suggested that PTMsnp can provide new perspectives on cancer studies, and subsequent experimental validation may help to discover novel mechanisms in cancerogenesis.

### PTMsnp Identifies Potential Disease Drivers in GWAS Dataset

**FIGURE 4:**
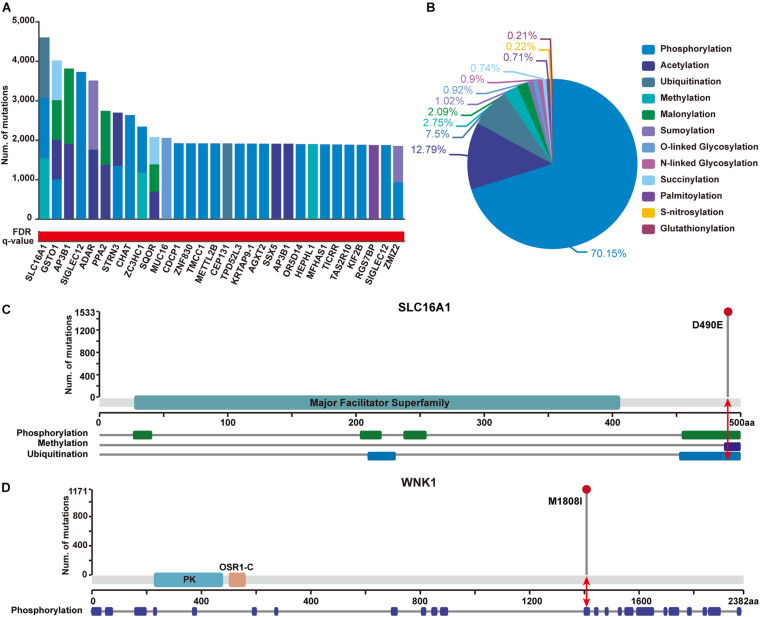
Significantly PTM-mutated proteins identified from a GWAS dataset of Type 2 Diabetes (T2D) samples with 1,916 individuals. **(A)** The top 30 genes ranked by the number of significant PTM-related mutations. Bar height shows the number of samples harboring mutations in each PTM type, respectively. The red and white gradient bar below represents the FDR *q*-value. **(B)** The proportion of PTM-related mutations of each modification type in identified proteins. **(C)** SLC16A1 has the most frequent PTM-related mutations affecting three types of modifications. Upper panel shows the number of mutated samples per position. Protein domain of SLC16A1 are shown in green region along the sequence. The modified regions of three PTM types on SLC16A1 protein are shown below. The modified position where the mutation has occurred is indicated by a red arrow. **(D)** Mutation M1808I were identified to significantly alter phosphorylation status of WNK1. Protein domain of WNK1 are shown in blue and orange (PK, Protein kinase domain; OSR1-C, Oxidative-stress-responsive kinase 1 C-terminal domain). The modified position where the mutation has occurred is indicated by a red arrow.

In addition, to show the practicability of applying PTMsnp in other disease-related studies, we further performed an analysis on a GWAS dataset of type 2 diabetes (T2D) samples from 1,916 tested individuals. Using PTMsnp, a total of 257 genes (Supplementary Table S5) with significant mutations across 12 different PTM types were identified (FDR *P* = 0.05, Figure 4A). More than 70% PTM-related mutations are located in phosphorylation regions (Figure 4B), which is reasonable when considering the broadness of phosphorylation sites. SLC16A1 has the most frequent PTM-related mutations affecting three types of modifications including phosphorylation, methylation, and ubiquitination (Figure 4C). The solute carrier family 16 member 1 (SLC16A1) gene, which encodes the monocarboxylate transporter 1 (MCT1) protein, is a proton-coupled monocarboxylate transporter catalyzing the transportation of many monocarboxylates, such as lactate and pyruvate, across cell membranes. Many studies have revealed that mutations on SLC16A1 are associated with abnormal insulin secretion (Pullen et al., 2012; Al-Khawaga et al., 2019). Moreover, Nikooie et al. (2013) have reported that the expression of MCT1 is dramatically reduced in diabetes, which may lead to increased insulin resistance. Besides, Zhao et al. (2001) have also found that the overexpression of MCT protein throughout the islet could involve in deranged insulin secretion in some type 2 diabetes. These studies suggested that the abnormal expression of MCT1 may be one of the pathogenic mechanisms of T2D. On the other hand, it has been reported that cAMP can cause the dephosphorylation of MCT1 and thereby reduce its surface expression (Smith et al., 2012). This evidence implies a positive synergy mechanism between MCT1 phosphorylation and its expression. Based on the existing literatures and our results, we speculated that our identified mutations on SLC16A1 can potentially affect its phosphorylation state, and may further lead to abnormal glucose sensing and even insulin resistance in T2D by changing the expression level of MCT1. Therefore, we can reasonably believe that SLC16A1 can serve as a novel PTM-mediated T2D driver genes.

Furthermore, 23 well-known T2D-related genes were found to carry significant PTM-related mutations in our analysis (Supplementary Table S6). Of these genes, With-no-lysine 1 (WNK1) kinase is taken here as an illustrative example (Figure 4D). WNK1 is serine-threonine kinase and highly expressed in skeletal muscles. An existing study has shown that insulin can phosphorylate WNK1, thereby activating glucose transporter 4 (GLUT4) translocation and stimulating glucose uptake through the PI3K/Akt signaling cascade. Decreased WNK1 phosphorylation were observed in T2D skeletal muscle, providing a new perspective on WNK1 function in T2D (Kim et al., 2018). Interestingly, we observed that the M1808I mutation on WNK1 was significantly enriched around the phrosphorylation site Thr1810 in T2D patients, implying a pathogenic role of WNK1 in T2D via its aberrant dephosphorylation. Given this observation, it is worthy to perform further experiments to verify the functional role of such mutation regarding to phrosphorylation process.

## Summary and Perspectives

Genetic mutations in human genomes include both driver mutations that provide selective advantages to disease progression and neutral passenger mutations present due to genome instability. A key challenge facing the biological community is to distinguish only a few driver mutations from the majority of passenger mutations. Previous studies have proven that combining mutations with other important functional features may provide extra guidance for driver event detection compared to traditional frequency-based methods. PTMs have been successfully used to predict driver mutations in diseases owing to their extensive functions in biological processes. However, the lack of an integrated resource of PTM sites as well as a user-friendly web interface greatly hindered the exploration of PTM-mediated disease progression. The PTMsnp web server was elaborately designed and dedicated for addressing such issues. With the collected PTM dataset, the vast majority of genetic mutations can be further annotated, and potential disease-driven genes can be inferred from the perspective of aberrant PTM status. As applications, we have successfully applied PTMsnp to the detection of cancer driver genes and disease-related genes from type 2 diabetes cohorts. This analysis revealed the prospect of using PTMsnp to explore the underlying pathogenesis of known disease-related mutations and to discover novel cancer drivers for further clinical research.

PTMsnp can be further enhanced in several aspects in the future. First, more genetic mutations such as population mutation datasets can be supported in future updates of PTMsnp. Different PTM processes can be orchestrated by different enzymatic systems, forming a dynamic regulatory cycle in normal cells. The perturbation of such a dynamic regulatory cycle may also lead to certain abnormalities. Therefore, the current algorithm can be further extended to consider mutations in PTM enzymes. In addition, the protein-protein interaction network may also be considered to interpret the impact of genetic mutations on PTM enzyme-substrate interactions, for example, kinase-substrate interactions in phosphorylation. With the ongoing database update and algorithm extensions, we expect PTMsnp to become a useful web server for the biomedical research community and to provide more valuable insights into disease biology and therapy development.

## Data Availability Statement

The original contributions presented in the study are included in the article/Supplementary Material, further inquiries can be directed to the corresponding authors.

## Author Contributions

DP performed data analysis, implemented the PTMsnp algorithm, and wrote the manuscript. HL and BH were, respectively, responsible for the front-end page display and back-end logic design of the PTMsnp website. HZ designed original website page. LC and SL manually collected PTM sites from published literatures. ZZ and YX guided the methodology of the research. JR was responsible for supervision, funding acquisition, and writing – review. YBX supervised this work, designed the PTMsnp algorithm, reviewed, and edited the manuscript. All authors have read and approved the manuscript.

## Conflict of Interest

The authors declare that the research was conducted in the absence of any commercial or financial relationships that could be construed as a potential conflict of interest.

## Footnotes

1 https://portal.gdc.cancer.gov/
