# Supplementary material for: PTMsnp: A Web Server for the Identification of Driver Mutations That Affect Protein Post-translational Modification
Source: Front Cell Dev Biol. 2020 Nov 10;8:593661. doi: 10.3389/fcell.2020.593661 (PMC7683509; doi:10.3389/fcell.2020.593661)
Supplement: Supplementary file 1 [file Data_Sheet_1.DOCX]

Supplementary Material

# Supplementary Methods

## Identification of PTM-related mutations

For each modification site, we defined the PTM motif as a flanking region with 7 amino acids upstream and downstream in which a particular PTM site was located at the central position. For each protein, we merged the motif region to construct a modification region. Correspondingly, the remaining sequences were merged separately and denoted as background regions. The mutations that located in the modification region are denoted as modification-related mutations, otherwise it will be regarded as modification-irrelevant mutations.

## Detection of PTM-related driver proteins by hierarchical Bayesian model

In our model, we hypothesized that mutations on the motif regions would probably damage the PTM modification process, thereby influencing the function of their corresponding proteins via PTM-related pathways. If such mutations are highly correlated with diseases progression, they will probably undergo strong positive selection during the disease development process, and therefore, unexpectedly high mutation rates will be observed in these regions. In view of this assumption, we can identify PTM modification-related driver proteins by comparing the mutation rates in both motif regions and modification-free regions. Accordingly, a null hypothesis that the mutation rate in the motif region is the same as which in the modification-free region is proposed.

For a given protein, we first counted up the number of genetic mutations in each motif region of a particular modification. This numbers were represented as $Y_{1},Y_{2},...,Y_{k}$. Similarly, mutations in each background region are counted and presented as$Y_{k+1},Y_{k+2},...,Y_{n}$. According to this definition, the observed counts *Y* can be described by a Poisson distribution as shown in equation 1.

$\begin{matrix} Y_{i}\sim Poisson(\lambda_{1})=\frac{\lambda^{Y_{\text{i}}}\text{e}^{\text{-}\lambda}}{Y_{i}!},i=1,2,...,k \\ Y_{i}\sim Poisson(\lambda_{2})=\frac{\lambda^{Y_{\text{i}}}\text{e}^{\text{-}\lambda}}{Y_{i}!},i=k+1,k+2,...,n \end{matrix}$ (1)

Where $\lambda_{1}$ and $\lambda_{2}$ represented the mutation rates of the motif and background region.

Due to sequencing bias, the mutation rate between different genomic positions will vary differently, and this bias may have a great effect on the model performance. Take this effect into consideration, we add a prior distribution on $\lambda_{1}$ and$\lambda_{2}$ to build a hierarchical model. Since the Gamma distribution is a conjugate prior for the Poisson distribution, two gamma distributions with different shape parameters $\alpha$ and scale parameters $\beta$ are used (Equation 2).

(2)

To compare the mutation rates in the modification and background regions, we first need to compute the posterior distribution of $\lambda_{1}$ and $\lambda_{2}$ given the observed data *Y* in our hierarchical model, *i.e.*, calculating $P(\lambda_{1}|Y)$ and $P(\lambda_{2}|Y)$. To achieve this goal, the full joint distribution of $\lambda_{1}$ and$\lambda_{2}$should be computed. According to Bayesian theory, the formula can be written as shown in equation 3.

$\begin{matrix} P(\lambda_{1},\lambda_{2}|Y)=\frac{P(Y|\lambda_{1},\lambda_{2})P(\lambda_{1},\lambda_{2})}{\sum P(Y|\lambda_{1},\lambda_{2})P(\lambda_{1},\lambda_{2})}\propto P(Y|\lambda_{1},\lambda_{2})P(\lambda_{1},\lambda_{2}) \\ =P(Y_{1:k}|\lambda_{1})P(\lambda_{1})P(Y_{k+1:n}|\lambda_{2})P(\lambda_{2}) \end{matrix}$ (3)

Where$P(Y|\lambda_{1},\lambda_{2})$ is the likelihood of $Y$. $P(\lambda_{1,}\lambda_{2})$is the prior distribution of$\lambda_{1}$ and$\lambda_{2}$. As we plugged the probability density function of Poisson and Gamma distribution into equation 3, we can obtain:

$\begin{matrix} P(\lambda_{1},\lambda_{2}|Y)=P(Y_{1:k}|\lambda_{1})P(\lambda_{1})P(Y_{k+1:n}|\lambda_{2})P(\lambda_{2}) \\ =\prod_{i=1}^{k} \frac{\lambda_{1}^{Y_{i}}e^{-\lambda_{1}}}{Y_{i}!}\frac{\beta_{1}^{\alpha_{1}}\lambda_{1}^{\alpha_{1}-1}e^{-\beta_{1}\lambda_{1}}}{\Gamma(\alpha_{1})}\prod_{i=k+1}^{n} \frac{\lambda_{2}^{Y_{i}}e^{-\lambda_{2}}}{Y_{i}!}\frac{\beta_{2}^{\alpha_{2}}\lambda_{2}^{\alpha_{2}-1}e^{-\beta_{2}\lambda_{2}}}{\Gamma(\alpha_{2})} \end{matrix}$ (4)

The standard procedure to calculate the posterior distribution from the above full joint probability requires integrating over other unrelated variables in Equation 4. However, this work can hardly be done by analytical method. Instead, it is much easier to estimate the posterior distributions by the Markov Chain Monte Carlo (MCMC) method, *i.e.* Gibbs sampling.

Implementation of Gibbs sampling requires the full-conditional probabilities of all variables, namely $P(\lambda_{1}|Y_{1:n},\lambda_{2})$ and $P(\lambda_{2}|Y_{1:n},\lambda_{1})$ in equation 4. We computed these two probabilities by taking the logarithm of equation 4:

$\begin{matrix} \ln P(\lambda_{1},\lambda_{2}|Y)=\ln(\prod_{i=1}^{k} \frac{\lambda_{1}^{Y_{i}}e^{-\lambda_{1}}}{Y_{i}!}\frac{\beta_{1}^{\alpha_{1}}\lambda_{1}^{\alpha_{1}-1}e^{-\beta_{1}\lambda_{1}}}{\Gamma(\alpha_{1})}\prod_{i=k+1}^{n} \frac{\lambda_{2}^{Y_{i}}e^{-\lambda_{2}}}{Y_{i}!}\frac{\beta_{2}^{\alpha_{2}}\lambda_{2}^{\alpha_{2}-1}e^{-\beta_{2}\lambda_{2}}}{\Gamma(\alpha_{2})}) \\ =\sum_{i=1}^{k} (Y_{i}\ln\lambda_{1}-\lambda_{1}-\ln Y_{i}!)+\alpha_{1}\ln\beta_{1}+(\alpha_{1}-1)\ln\lambda_{1}-\beta_{1}\lambda_{1}-\ln\Gamma(\alpha_{1}) \\ +\sum_{i=k+1}^{n} (Y_{i}\ln\lambda_{2}-\lambda_{2}-\ln Y_{i}!)+\alpha_{2}\ln\beta_{2}+(\alpha_{2}-1)\ln\lambda_{2}-\beta_{2}\lambda_{2}-\ln\Gamma(\alpha_{2}) \\ =-k\lambda_{1}+\ln\lambda_{1}\sum_{i=1}^{k} Y_{i}-\sum_{i=1}^{k} \ln Y_{i}!-(n-k)\lambda_{2}+\ln\lambda_{2}\sum_{i=k+1}^{n} Y_{i}-\sum_{i=k+1}^{n} \ln Y_{i}! \\ +\alpha_{1}\ln\beta_{1}+(\alpha_{1}-1)\ln\lambda_{1}-\beta_{1}\lambda_{1}-\ln\Gamma(\alpha_{1})+\alpha_{2}\ln\beta_{2}+(\alpha_{2}-1)\ln\lambda_{2}-\beta_{2}\lambda_{2}-\ln\Gamma(\alpha_{2}) \end{matrix}$ (5)

The full-conditional probabilities for a given variable can be derived by abstracting out only those items containing the interested variable from the joint probability and treating other components as constants. Based on this rule, the full conditional posterior probability of$\lambda_{1}$and$\lambda_{2}$can be calculated as formulas below,

$\begin{matrix} \ln P(\lambda_{1}|Y_{1:n},\lambda_{2})\text{=}\ln P(\lambda_{1}|Y_{1:k},\lambda_{2}) \\ =-k\lambda_{1}+\ln\lambda_{1}\sum_{i=1}^{k} Y_{i}+(\alpha_{1}-1)\ln\lambda_{1}-\beta_{1}\lambda_{1} \\ =(\sum_{i=1}^{k} Y_{i}+\alpha_{1}-1)\ln\lambda_{1}-(k+\beta_{1})\lambda_{1} \\ \propto\ln Gamma(\sum_{i=1}^{k} Y_{i}+\alpha_{1},k+\beta_{1}) \end{matrix}$ (6)

$\begin{matrix} \ln P(\lambda_{2}|Y_{1:n},\lambda_{1})\text{=}\ln P(\lambda_{2}|Y_{k+1:n},\lambda_{1}) \\ =-(n-k)\lambda_{2}+\ln\lambda_{2}\sum_{i=k+1}^{n} Y_{i}+(\alpha_{2}-1)\ln\lambda_{2}-\beta_{2}\lambda_{2} \\ =(\sum_{i=k+1}^{n} Y_{i}+\alpha_{2}-1)\ln\lambda_{2}-(n-k+\beta_{2})\lambda_{2} \\ \propto\ln Gamma(\sum_{i=k+1}^{n} Y_{i}+\alpha_{2},n-k+\beta_{2}) \end{matrix}$ (7)

Equation 6 and 7 demonstrated that the full-conditional probabilities of$\lambda_{1}$and$\lambda_{2}$can finally reduce to gamma distribution. The full-conditional distribution can be directly sampled from the distribution below,

$P(\lambda_{1}|Y_{1:n},\lambda_{2})=Gamma(\sum_{i=1}^{k} Y_{i}+\alpha_{1},k+\beta_{1})$ (8)

$P(\lambda_{2}|Y_{1:n},\lambda_{1})\text{=}Gamma(\sum_{i=k+1}^{n} Y_{i}+\alpha_{2},n-k+\beta_{2})$ (9)

To test the difference between the mutation rates of background region and modification region, a variable of interest might be the relative mutation rate defined as$R=\frac{\lambda_{1}}{\lambda_{2}}$. According to the published method (Carlin et al., 1992), if a variable *W* actually appears as a function of another variable *U*, the full-conditional probability of *W* can be obtained by univariate transformation from that of *U*. Following these mathematicalrule, we further transform$\lambda_{1}$to$R$to obtain the full-conditional probability of$R$.

$\lambda_{1}=R\lambda_{2}$ (10)

Plugging equation 10 into equation (6),

$\begin{matrix} \ln P(R|Y_{1:n},\lambda_{1},\lambda_{2})=\ln P(R\lambda_{2}|Y_{1:n},\lambda_{2}) \\ =-kR\lambda_{2}+\ln R\lambda_{2}\sum_{i=1}^{k} Y_{i}+(\alpha_{1}-1)\ln R\lambda_{2}-\beta_{1}R\lambda_{2} \\ =-kR\lambda_{2}+(\ln R+\ln\lambda_{2})\sum_{i=1}^{k} Y_{i}+(\alpha_{1}-1)(\ln R+\ln\lambda_{2})-\beta_{1}R\lambda_{2} \end{matrix}$ (11)

Again, using the mathematical rule presented above, the full-conditional probability of$R$can be derived as:

$\begin{matrix} \ln P(R|Y_{1:n},\lambda_{1},\lambda_{2})\propto-kR\lambda_{2}+\ln R\sum_{i=1}^{k} Y_{i}+(\alpha_{1}-1)\ln R-\beta_{1}R\lambda_{2} \\ =(\sum_{i=1}^{k} Y_{i}+\alpha_{1}-1)\ln R-(k\lambda_{2}+\beta_{1}\lambda_{2})R \\ \propto\ln Gamma(\sum_{i=1}^{k} Y_{i}+\alpha_{1},k\lambda_{2}+\beta_{1}\lambda_{2}) \end{matrix}$ (12)

Thus, the full-conditional probability of$R$can also be reduced to a gamma distribution,

$P(R|Y_{1:n},\lambda_{1},\lambda_{2})=Gamma(\sum_{i=1}^{k} Y_{i}+\alpha_{1},k\lambda_{2}+\beta_{1}\lambda_{2})$ (13)

After calculating all the full conditional probabilities of each variable, we can now use Gibbs sampling algorithm to sample from Equation 8, 9 and 13 to estimate the posterior distribution of these parameters. During the sampling process, we performed 10,000 iterations in total and removed the first 2,000 iterations as a burn-in process. Details of the Gibbs sampling algorithm is described below,

**Gibbs sampling in hierarchical Bayesian model**

Initialize $\lambda_{1},\lambda_{2},R$

**For** iteration$t=1,2,...$**do**

$$\begin{matrix} \lambda_{1}^{(t)}\sim Gamma(\sum_{i=1}^{k} Y_{i}+\alpha_{1},k+\beta_{1}) \\ \lambda_{2}^{(t)}\sim Gamma(\sum_{i=k+1}^{n} Y_{i}+\alpha_{2},n-k+\beta_{2}) \\ R^{(t)}\sim Gamma(\sum_{i=1}^{k} Y_{i}+\alpha_{1},k\lambda_{2}^{t}+\beta_{1}\lambda_{2}^{t}) \end{matrix}$$

**End for**

By taking$\alpha_{1}=\alpha_{2}=1.0$and$\beta_{1}=\beta_{2}=0.5$, we can get 8,000 sampling result during Gibbs sampling process. According to our original hypothesis of modification-related driver proteins, the comparison of mutation rates in motif and background regions can be simplified to the significant test of$R$.

$\begin{matrix} H_{0}:R\leq1 \\ H_{1}:R>1 \end{matrix}$ (14)

The p-value under the null hypothesis is then calculated from the posterior distribution of$R$. For each tested protein, the probability of observing the relative mutation rate less than 1 can be calculated. To control false positive rate, the Benjamini-Hochberg procedure is applied to each p-value. If the corrected p-value for a given protein is lower than the significant level, *i.e.* 0.05, we will identify it as a significantly mutated protein.

# Supplementary Tables

**Supplementary Table 1.** Statistics of modification sites from 33 PTM types and somatic mutations across 33 different cancer types included in PTMsnp web server.

**Supplementary Table 2.** Significantly PTM-mutated proteins identified in TCGA cohorts of 33 cancer types.

**Supplementary Table 3.** Significantly PTM-related mutations identified in TCGA cohorts of 33 cancer types.

**Supplementary Table 4.** PTMs with significant mutations in known cancer genes identified by PTMsnp.

**Supplementary Table 5.** A list of significantly PTM-mutated proteins that identified from a GWAS dataset of Type 2 Diabetes (T2D) samples with 1,916 individuals.

**Supplementary Table 6.** Known T2D-related genes/proteins curated in the Harmonizome (Rouillard et al., 2016) database. For genes/proteins from gene-disease association studies, only those with a p-value less than 0.05 are retained. The known T2D-related genes identified by PTMsnp is marked with yellow.

# Supplementary References

Carlin, B.P., Gelfand, A.E., and Adrian, F.M.S. (1992). Hierarchical Bayesian Analysis of Changepoint Problems. *Journal of the Royal Statistical Society. Series C (Applied Statistics)* 41(2)**,** 389-405. doi: 10.2307/2347570.

Rouillard, A.D., Gundersen, G.W., Fernandez, N.F., Wang, Z., Monteiro, C.D., McDermott, M.G., et al. (2016). The harmonizome: a collection of processed datasets gathered to serve and mine knowledge about genes and proteins. *Database : the journal of biological databases and curation* 2016**,** baw100. doi: 10.1093/database/baw100.
